# Evaluation of Serum Sestrin 2 Levels in Patients Diagnosed with Endometrial Polyps and Uterine Leiomyomas

**DOI:** 10.3390/jcm13123413

**Published:** 2024-06-11

**Authors:** Teymur Bornaun, Selim Akkaya, Hamid Zafer Güven

**Affiliations:** 1 Department of Obstetrics and Gynecology, Istanbul University Health Sciences Istanbul Bagcilar Training and Research Hospital, Istanbul 34200, Turkey; 2 Istanbul University Health Sciences Istanbul Bagcilar Training and Research Hospital, Istanbul 34200, Turkey; drakkaya.selim@gmail.com (S.A.); hzguven@hotmail.com (H.Z.G.)

**Keywords:** sestrin 2, endometrial polyp, leiomyoma

## Abstract

**Background/Objectives:** This study investigates the correlation between the serum levels of Sestrin 2 and the presence of endometrial polyps or uterine leiomyomas, aiming to enhance the understanding of the pathophysiology underlying these gynecological conditions and evaluate the potential of Sestrin 2 as an early diagnostic biomarker. **Methods:** In a prospective case-control format, patients with preliminary diagnoses of endometrial polyps or uterine leiomyomas confirmed by histopathological analysis following surgery were included. This study analyzed serum Sestrin 2 levels across different patient groups, revealing significant variations that underscore the diagnostic value of Sestrin 2. **Results:** Elevated serum Sestrin 2 levels were observed in patients with endometrial polyps and uterine leiomyomas compared to the control group, suggesting its utility as a novel marker for early detection. **Conclusions:** The study indicates the promising role of serum Sestrin 2 levels as a valuable biomarker for early diagnosis of endometrial polyps and uterine leiomyomas, advocating for further research into its diagnostic and therapeutic potential.

## 1. Introduction

Endometrial polyps and uterine leiomyomas are among the most common benign gynecological conditions affecting women worldwide. Sestrin 2 is highly expressed in organs like kidneys, lungs, leukocytes, liver, gastrointestinal tract, and brain. Additionally, the increased levels of Sestrin 2 have been associated with other gynecological pathologies such as PCOS and endometrial cancer. These entities not only contribute significantly to morbidity, such as abnormal uterine bleeding and infertility, but also pose challenges in diagnosis and management [1,2]. Endometrial polyps, characterized by localized hyperplastic growth of glandular epithelium and stroma, and leiomyomas, originating from myometrial smooth muscle cells, albeit histologically distinct, share common etiological and clinical profiles [3]. The pathogenesis of these conditions has been linked to oxidative stress and inflammatory pathways, underscoring the importance of identifying biomarkers reflective of these processes [4,5]. Sestrin 2 is a crucial protein in cellular stress response and homeostasis, linked to oxidative stress and inflammatory pathways. These pathways are significant in the pathogenesis of endometrial polyps and uterine leiomyomas.

In recent years, Sestrin 2, a protein integral to the cellular response to stress and homeostasis, has emerged as a potential biomarker for various diseases, including those affecting the female reproductive system [6]. Its role in modulating oxidative stress, inflammation, and cell proliferation makes it a promising candidate for studying its association with gynecological conditions like endometrial polyps and uterine leiomyomas [7]. The present study endeavors to explore the relationship between serum Sestrin 2 levels and these conditions, aiming to unravel its pathophysiological relevance and assess its utility in early diagnosis.

The exploration of Sestrin 2’s involvement in gynecological pathologies is anchored in its well-documented functions across different stress conditions and disease states, providing a novel perspective on the mechanistic underpinnings of endometrial polyps and uterine leiomyomas [8,9]. By investigating the correlation between the serum levels of Sestrin 2 and the presence of these conditions, this study contributes to a deeper understanding of their pathophysiology and explores the potential of Sestrin 2 as a diagnostic biomarker, opening avenues for targeted therapeutic interventions [10].

## 2. Materials and Methods

### 2.1. Study Design

This research was conducted as a prospective, case-control study at the Health Sciences University Bağcılar Training and Research Hospital’s Gynecology Clinic. Its aim was to explore the contribution of serum Sestrin 2 levels to the etiology of endometrial polyps and uterine leiomyomas, their diagnostic value as a non-invasive method, and the potential implications for future treatments. Ethical approval was obtained from the Clinical Research Ethics Committee of Health Sciences University Bakırköy Dr. Sadi Konuk Training and Research Hospital on 6 February 2023 (Approval No: 2023-03-08).

### 2.2. Participants

Participants were enrolled from individuals visiting the Gynecology Outpatient Clinic with preliminary diagnoses of endometrial polyps or uterine leiomyomas via transvaginal ultrasonography. Laboratory tests were conducted to exclude these diagnoses before enrolling patients in the study groups. Those confirmed to have these conditions postoperatively (hysteroscopic resection or laparotomy) were included in two distinct groups. The control group comprised individuals without these conditions as confirmed by ultrasonography. In total, 60 patients with endometrial polyps, 57 with uterine leiomyomas, and 59 control individuals were recruited. Comprehensive informed consent was obtained from all participants.

#### Inclusion and Exclusion Criteria

The inclusion criteria encompassed women aged 18–50 years with confirmed diagnoses of endometrial polyps or uterine leiomyomas and no additional health conditions. The exclusion criteria were as follows: under 18 years of age, postmenopausal status, diagnosis of Polycystic Ovary Syndrome (according to Rotterdam criteria), history of blood transfusion within the last six months, suspected or confirmed malignancy, chronic medical conditions, recent or active infections, pregnancy, usage of medications known to influence Sestrin 2 levels, and patients with type 2 diabetes or dyslipidemia, as these conditions are known to affect Sestrin 2 levels.

### 2.3. Sample Collection and Processing

Blood samples were collected into biochemistry tubes (BD Vacutainer, Becton Dickinson, Plymouth, UK), allowed to coagulate for 15 min, then centrifuged at 3000 rpm for 10 min. The serum was then aliquoted into labeled Eppendorf tubes and stored at −80 °C until analysis.

#### Sestrin 2 Assay

The serum Sestrin 2 levels were quantified using the Human Sestrin 2 ELISA kit (BT LAB, Cat. No. E3437Hu). The protocol was conducted at room temperature, beginning with the reconstitution of standards and samples, followed by the sequential addition of reagents, incubation, and detection phases as specified by the manufacturer’s guidelines. The kit has a sensitivity of 90% and a specificity of 95%.

### 2.4. Statistical Analysis

The data were subjected to the Shapiro–Wilk test for a normality assessment. One-way ANOVA and Kruskal–Wallis tests were applied for parametric and non-parametric variables, respectively. The relationships between qualitative variables were examined using the likelihood ratio chi-squared and Pearson’s chi-squared tests. The association between the Sestrin 2 levels and the independent variables was analyzed using Firth’s penalized maximum likelihood estimation. The diagnostic accuracy was evaluated using the area under the ROC curve (AUC), sensitivity, specificity, and positive and negative predictive values. All analyses were performed using the SPSS software package (Version 29) and the SAS software package (Version 9.4). Additionally, the Systemic Immune-Inflammation Index (SII) was calculated using the following formula:

$$\mathrm{SII}=\frac{\mathrm{Platelet}\;\mathrm{Count}\;\times \;\mathrm{Neutrophil}\;\mathrm{Count}}{\mathrm{Lymphocyte}\;\mathrm{Count}}$$

This index is used to evaluate the balance between inflammation and immune response and can be a useful marker in various conditions.

## 3. Results

The results section delineates the comprehensive analysis of demographic and clinical characteristics of the participants, alongside the evaluation of the serum Sestrin 2 levels across the different groups. Statistical significance and interpretative insights are drawn from the comparative analyses.

### 3.1. Demographic and Clinical Characteristics

Our study included three distinct groups: a control group (n = 59), an endometrial polyp group (n = 60), and a uterine leiomyoma (myoma) group (n = 57). Statistical analyses revealed significant demographic and clinical variances among these groups.

Table 1 presents a comprehensive overview of the demographic and clinical characteristics, including age, BMI, gravidity, parity, and smoking habits among the control, polyp, and myoma groups. Statistical analysis revealed a significant age difference among these groups, whereas other demographic factors remained consistent. The distribution of clinical complaints, such as infertility, incontinence, menorrhagia, and pelvic pain, across the study groups, highlighted significant differences in the prevalence of these symptoms among the groups (*p* < 0.01).

The serum Sestrin 2 values for the three groups of patients are included in Table 1 for clarity. Various non-significant correlations with age, BMI, lesion size, gravidity, and parity were observed. This indicates a significant elevation of the Sestrin 2 levels in the polyp and myoma groups compared to the control group. Statistical analysis supports the significance of these findings, reinforcing the potential diagnostic value of Sestrin 2 levels in these conditions.

### 3.2. Correlation Analysis

In-depth correlation analysis was aimed at discerning any significant associations between the Sestrin 2 levels and various clinical parameters including age, BMI, lesion size (for both polyps and myomas), gravidity, and parity. No significant correlation was found between the Sestrin 2 levels and demographic characteristics such as age, BMI, or the size of polyps and myomas, suggesting that the elevation of Sestrin 2 levels may be independent of these factors (Table 2).

Table 2 explores the correlation between Sestrin 2 levels and various clinical parameters, including age, BMI, polyp size, and myoma size. Statistical tests indicated no significant correlations, reinforcing the hypothesis that Sestrin 2 elevation is specifically related to the presence of polyps and myomas rather than to general demographic characteristics.

### 3.3. Diagnostic Performance of Sestrin 2

**Figure 1:**
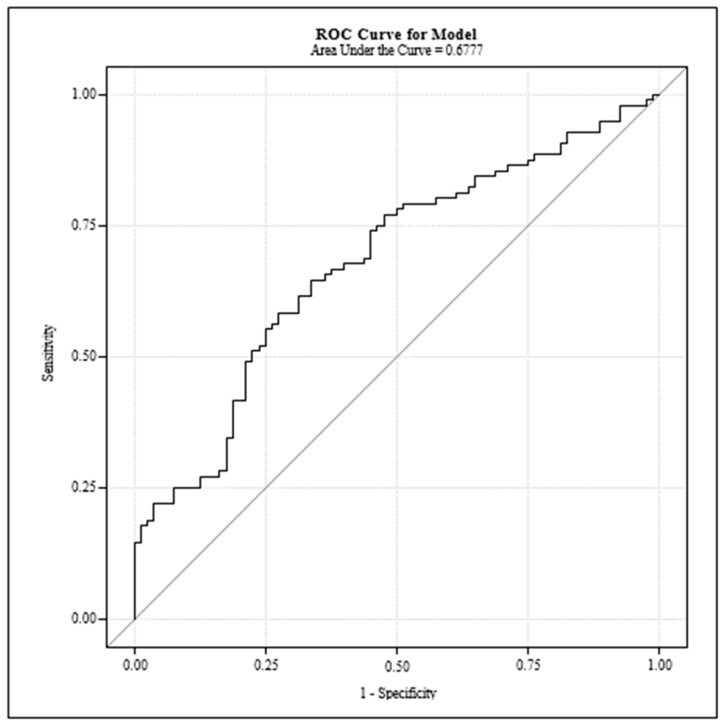
Receiver operating characteristics (ROC) curve for Estrin 2 levels.

The diagnostic utility of Sestrin 2 levels in distinguishing between healthy individuals and those with polyps or myomas was further evaluated using Receiver Operating Characteristic (ROC) curve analysis (Figure 1).

Figure 1 illustrates the ROC curve derived from the Sestrin 2 level analysis, aimed at evaluating the biomarker’s effectiveness in discriminating between the study groups. The Receiver Operating Characteristic (ROC) curve analysis for Sestrin 2 levels demonstrates the diagnostic sensitivity and specificity of Sestrin 2 as a biomarker for detecting uterine polyps and myomas. Furthermore, the area under the curve (AUC) indicates a moderate diagnostic ability.

### 3.4. Statistical Significance and Clinical Implications

The statistical analysis underscores a pivotal finding—the elevated Sestrin 2 levels are significantly associated with the presence of endometrial polyps and uterine myomas. While no discernible correlation was found with the demographic and clinical characteristics, the notable difference in Sestrin 2 levels between the control and affected groups points to its potential role as a non-invasive biomarker for these conditions.

The lack of correlation with lesion size and other parameters suggests that while Sestrin 2 can be indicative of pathology, it might not reflect the severity or the scale of the condition. This aspect opens avenues for future research, focusing on the mechanistic link between Sestrin 2 and uterine pathologies and exploring its potential utility in monitoring disease progression or response to treatment.

## 4. Discussion

The results of our study indicate that serum Sestrin 2 protein levels are approximately 1.10 ng/mL in the control group, 1.94 ng/mL in the myoma group, and 1.91 ng/mL in the polyp group, with a statistically significant difference observed across the groups (*p* < 0.05). These findings align with the prior research that underscores the diagnostic potential of Sestrin 2 levels in detecting uterine pathologies [11,12]. The elevation of Sestrin 2 in the myoma and polyp groups, in contrast with the control group, highlights its significance as a biomarker, potentially paving the way for non-invasive diagnostics and treatment strategies [13].

The exact causes of endometrial polyps remain elusive, although they are believed to stem from a combination of genetic, inflammatory, hormonal, and iatrogenic factors. A majority of the cases are identified in individuals aged between 40 and 49, which is consistent with the mean age of 43 for patients with endometrial polyps in our study and fits within the age range reported in the previous literature [14]. This age correlation supports the existing hypothesis that hormonal changes during the perimenopausal period may play a crucial role in the development of polyps [15].

Similarly, the pathophysiology of uterine leiomyomas (myomas) is not entirely understood. However, studies have supported the role of oxidative stress and inflammation in the formation of fibroids [6,7]. A large-scale study conducted in the United States between 1989 and 1993 with over 95,000 women indicated that the prevalence rates of myomas per 1000 women per year increased with age. This aligns with our findings, where the mean age for patients with uterine myomas was 42, which is consistent with the literature [16]. These findings suggest that age-related oxidative stress might be a contributing factor in the development of myomas [17,18,19,20].

Our study further explores the relationship between Sestrin 2 levels and various demographic and clinical characteristics, such as age, BMI, and lesion size, finding no significant correlation, which corroborates the findings from other studies thus indicating that Sestrin 2 levels do not significantly vary with these parameters [21,22,23,24,25]. This suggests that while Sestrin 2 can serve as an indicator of uterine pathology, it may not directly reflect the condition’s severity or progression. The lack of correlation with BMI is particularly interesting, given that obesity is known to contribute to chronic, low-grade inflammation and oxidative stress [26,27].

In addition, previous studies have demonstrated that oxidative stress biomarker levels are higher in obese individuals [28,29]. Our study observed no significant difference in BMI across the patient and control groups, suggesting that this parameter might not significantly influence Sestrin 2 levels, aligning with the previous research that found no significant relationship between Sestrin 2 levels and BMI [30,31]. This reinforces the notion that while Sestrin 2 levels are elevated in specific uterine conditions, they do not vary significantly with body mass index.

In summary, our study supports the potential of Sestrin 2 as a biomarker for uterine pathologies, particularly in differentiating between normal and pathological conditions such as myomas and polyps. Future research should focus on further elucidating the mechanisms underlying Sestrin 2 regulation and its potential role in non-invasive diagnostic strategies.

Overall, these findings contribute to the growing body of evidence supporting the use of Sestrin 2 as a valuable biomarker for the detection and monitoring of uterine pathologies. Future studies should focus on longitudinal analyses to determine the potential predictive value of Sestrin 2 levels in the progression of uterine diseases. Additionally, exploring the molecular mechanisms underlying Sestrin 2 regulation could provide deeper insights into its role in uterine health and disease, potentially leading to new therapeutic targets. Our study highlights the importance of integrating Sestrin 2 measurement into clinical practice to enhance the diagnostic accuracy and management of uterine conditions.

## 5. Study Limitations

At the end of our study, it is essential to address its limitations. The primary limitation is the exclusion of patients with associated conditions like type 2 diabetes or dyslipidemia, which could influence Sestrin 2 levels. Another limitation is the relatively small sample size, which may affect the generalizability of our findings. Further studies with larger cohorts are needed to validate these results.

## 6. Conclusions

Our study provides compelling evidence that serum Sestrin 2 levels are markedly elevated in patients with uterine myomas and endometrial polyps, compared to a control group, suggesting a significant role for Sestrin 2 in the pathology of these conditions. The findings reinforce the potential utility of Sestrin 2 as a non-invasive biomarker for the detection of uterine pathologies, offering a promising avenue for early diagnosis and the development of targeted therapeutic strategies.

While our analysis did not reveal a significant correlation between Sestrin 2 levels and demographic or clinical characteristics such as age, BMI, lesion size, gravidity, parity, or smoking status, it highlights the specificity of Sestrin 2 elevation in relation to uterine myomas and endometrial polyps. This specificity underlines the potential of Sestrin 2 to serve as a distinct marker for these conditions, independent of other patient characteristics.

The lack of correlation with lesion size and the absence of a significant difference in Sestrin 2 levels between myoma and polyp groups may indicate that Sestrin 2 elevation is a general response to uterine pathology rather than a marker of disease severity or type. These findings suggest that while Sestrin 2 can be an indicator of pathology presence, it might not be indicative of the pathology’s extent or progression.

In conclusion, our study underscores the importance of Sestrin 2 in the context of gynecological pathologies and supports further investigation into its diagnostic and therapeutic potential.

Future research should focus on elucidating the mechanisms through which Sestrin 2 is involved in the development of uterine pathologies and exploring its utility in monitoring disease progression or treatment response. Additionally, studying Sestrin 2 in a broader spectrum of gynecological conditions could further define its role and utility in gynecological oncology and beyond.

## Figures and Tables

**Table 1:** Comparative analysis of serum Sestrin 2 levels among study groups.

Categories	Control (n = 59)	Polyp (n = 60)	Myoma (n = 57)	*p* Values
Age (Mean)	37	43	42	*p* < 0.05
BMI (kg/m^2^)	26	27	27	*p* > 0.304
Gravidity	3	3	3	*p* > 0.463
Parity	2	2	3	*p* > 0.401
Smoking Yes, n (%)	26 (%35)	25 (%34)	23 (%31)	*p* > 0.919
Smoking No, n (%)	33 (%33)	35 (%34)	34 (%33)	-
Mean Sestrin 2 (ng/mL)	1.10097	1.94128	1.91240	*p* < 0.05

**Table 2:** Correlation coefficients between serum Sestrin 2 levels and clinical parameters.

Parameter	Correlation Coefficient (r)	*p* Value
Age	−0.061	0.519
BMI (kg/m^2^)	0.049	0.623
Lesion Size (cm)	−0.030 (Polyps)	0.765
	−0.024 (Myomas)	0.802
Gravidity	0.034	0.711
Parity	−0.050	0.598

## Data Availability

The authors confirm that the data supporting the findings of this study are available within the article.

## References

[1] Nijkang N.P., Anderson L., Markham R., Manconi F. (2019). Endometrial polyps: Pathogenesis, sequelae and treatment. SAGE Open Med..

[2] Salim S., Won H., Nesbitt-Hawes E., Campbell N., Abbott J. (2011). Diagnosis and Management of Endometrial Polyps: A Critical Review of the Literature. J. Minim. Invasive Gynecol..

[3] Agaoglu M.O., Agaoglu Z., Yucel K.Y., Ozturk F.H., Caglar T. (2023). Evaluation of maternal serum sestrin-2 levels in intrauterine growth restriction. Ir. J. Med. Sci..

[4] Wright J.D., Herzog T.J., Tsui J., Ananth C.V., Lewin S.N., Lu Y.-S., Neugut A.I., Hershman D.L. (2013). Nationwide trends in the performance of inpatient hysterectomy in the United States. Obstet Gynecol..

[5] Rivera C.M., Grossardt B.R., Rhodes D.J., Brown R.D.J., Roger V.L., Melton L.J.I., Rocca W.A. (2009). Increased cardiovascular mortality after early bilateral oophorectomy. Menopause.

[6] Rocca W.A., Bower J.H., Maraganore D.M., Ahlskog J.E., Grossardt B.R., de Andrade M., Melton L.J. (2007). Increased risk of cognitive impairment or dementia in women who underwent oophorectomy before menopause. Neurology.

[7] Rivera C.M., Grossardt B.R., Rhodes D.J., Rocca W.A. (2009). Increased mortality for neurological and mental diseases following early bilateral oophorectomy. Neuroepidemiology.

[8] Melton L.J., Khosla S., Malkasian G.D., Achenbach S.J., Oberg A.L., Riggs B.L. (2003). Fracture risk after bilateral oophorectomy in elderly women. J. Bone Miner. Res..

[9] Rocca W.A., Grossardt B.R., de Andrade M., Malkasian G.D., Melton L.J. (2006). Survival patterns after oophorectomy in premenopausal women: A population-based cohort study. Lancet Oncol..

[10] Tsilioni I., Filippidis A.S., Kerenidi T., Budanov A.V., Zarogiannis S.G., Gourgoulianis K.I. (2016). Sestrin-2 is significantly increased in malignant pleural effusions due to lung cancer and is potentially secreted by pleural mesothelial cells. Clin. Biochem..

[11] Indraccolo U., Di Iorio R., Matteo M., Corona G., Greco P., Indraccolo S.R. (2013). The pathogenesis of endometrial polyps: A systematic semi-quantitative review. Eur. J. Gynaecol. Oncol..

[12] Baird D.D., Dunson D.B., Hill M.C., Cousins D., Schectman J.M. (2003). High cumulative incidence of uterine leiomyoma in black and white women: Ultrasound evidence. Am. J. Obstet. Gynecol..

[13] Protic O., Toti P., Islam S., Occhini R., Giannubilo S.R., Catherino W.H., Cinti S., Petraglia F., Ciavattini A., Castellucci M. (2016). Possible involvement of inflammatory/reparative processes in the development of uterine fibroids. Cell Tissue Res..

[14] Santulli P., Borghese B., Lemaréchal H., Leconte M., Millischer A.-E., Batteux F., Chapron C., Borderie D. (2013). Increased Serum Oxidative Stress Markers in Women with Uterine Leiomyoma. PLoS ONE.

[15] Toyokuni S., Okamoto K., Yodoi J., Hiai H. (1995). Persistent oxidative stress in cancer. FEBS Lett..

[16] Pejic S., Kasapovic J., Todorovic A., Stojiljkovic V., Pajovic S.B. (2006). Lipid peroxidation and antioxidant status in blood of patients with uterine myoma, endometrial polypus, hyperplastic and malignant endometrium. Biol. Res..

[17] Budanov A.V., Karin M. (2008). p53 Target Genes Sestrin1 and Sestrin2 Connect Genotoxic Stress and mTOR Signaling. Cell.

[18] AlAshqar A., Lulseged B., Mason-Otey A., Liang J., Begum U.A.M., Afrin S. (2023). Oxidative Stress and Antioxidants in Uterine Fibroids: Pathophysiology and Clinical Implications. Antioxidants.

[19] Pasha M., Eid A.H., Eid A.A., Gorin Y., Munusamy S. (2017). Sestrin2 as a Novel Biomarker and Therapeutic Target for Various Diseases. Oxid. Med. Cell. Longev..

[20] Budanov A.V., Lee J.H., Karin M. (2010). Stressin’ Sestrins take an aging fight. EMBO Mol. Med..

[21] Kim M.J., Bae S.H., Ryu J.C., Kwon Y., Oh J.H., Kwon J., Moon J.S., Kim K., Miyawaki A., Lee M.G. (2016). SESN2/sestrin2 suppresses sepsis by inducing mitophagy and inhibiting NLRP3 activation in macrophages. Autophagy.

[22] Wang J., Tang Y., Zhang J., Wang J., Xiao M., Lu G., Li J., Liu Q., Guo Y., Gu J. (2022). Cardiac SIRT1 ameliorates doxorubicin-induced cardiotoxicity by targeting sestrin 2. Redox Biol..

[23] Gao A., Li F., Zhou Q., Chen L. (2020). Sestrin2 as a potential therapeutic target for cardiovascular diseases. Pharmacol. Res..

[24] Qu J., Luo M., Zhang J., Han F., Hou N., Pan R. (2021). A paradoxical role for sestrin 2 protein in tumor suppression and tumorigenesis. Cancer Cell Int..

[25] Wei J.L., Fang M., Fu Z.X., Zhang S., Guo J.B., Wang R. (2017). Sestrin 2 suppresses cells proliferation through AMPK/mTORC1 pathway activation in colorectal cancer. Oncotarget.

[26] Ro S.H., Xue X., Ramakrishnan S.K., Cho C.S., Namkoong S., Jang I., Semple I.A., Ho A., Park H.W., Shah Y.M. (2016). Tumor suppressive role of sestrin2 during colitis and colon carcinogenesis. eLife.

[27] Liang Y., Zhu J., Huang H., Xiang D., Li Y., Zhang D. (2016). SESN2/sestrin 2 induction-mediated autophagy and inhibitory effect of isorhapontigenin (ISO) on human bladder cancers. Autophagy.

[28] Chen K.B., Xuan Y., Shi W.J., Chi F., Xing R., Zeng Y.C. (2016). Sestrin2 expression is a favorable prognostic factor in patients with non-small cell lung cancer. Am. J. Transl. Res..

[29] Zhao B., Shah P., Budanov A.V., Qiang L., Ming M., Aplin A., Sims D.M., He Y.-Y. (2014). Sestrin2 Protein Positively Regulates AKT Enzyme Signaling and Survival in Human Squamous Cell Carcinoma and Melanoma Cells. J. Biol. Chem..

[30] Chae H.S., Gil M., Saha S.K., Kwak H.J., Park H.-W., Vellingiri B., Cho S.-G. (2020). Sestrin2 Expression Has Regulatory Properties and Prognostic Value in Lung Cancer. J. Pers. Med..

[31] Byun J.-K., Choi Y.-K., Kim J.-H., Jeong J.Y., Jeon H.-J., Kim M.-K., Hwang I., Lee S.-Y., Lee Y.M., Lee I.-K. (2017). A Positive Feedback Loop between Sestrin2 and mTORC2 Is Required for the Survival of Glutamine-Depleted Lung Cancer Cells. Cell Rep..

